# Supplementary material for: Development of actionable targets of multi-kinase inhibitors (AToMI) screening platform to dissect kinase targets of staurosporines in glioblastoma cells
Source: Sci Rep. 2022 Aug 13;12:13796. doi: 10.1038/s41598-022-18118-7 (PMC9376105; doi:10.1038/s41598-022-18118-7)

Figure S1

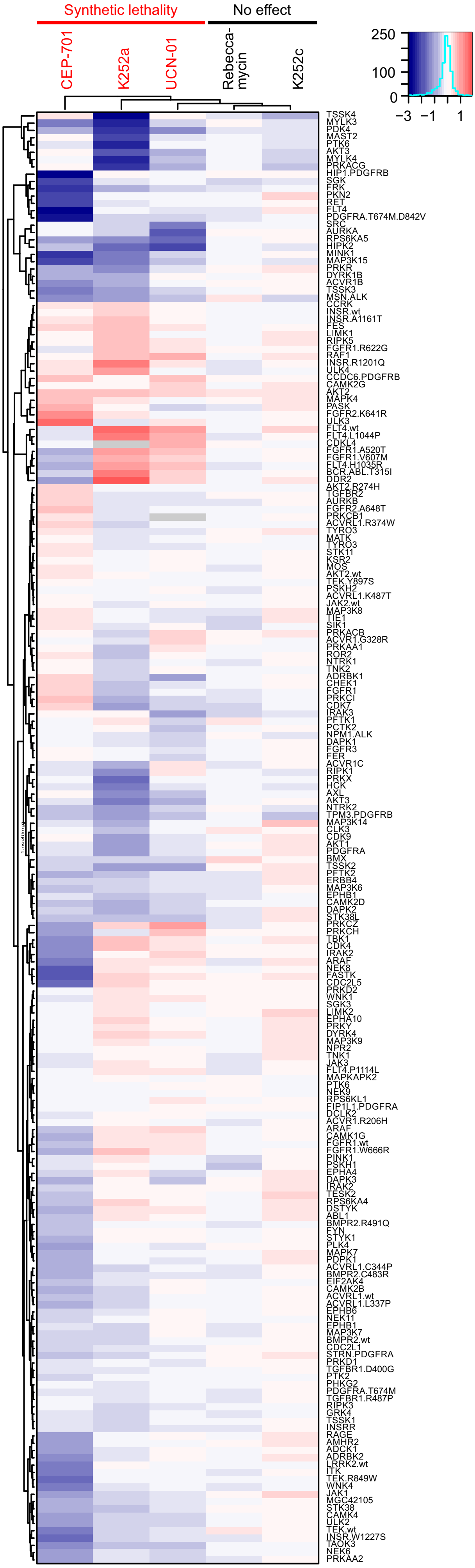

Figure S1. A high-resolution version of Figure 1C showing interaction of STS derivatives with kinases by Chaperone interaction assay. Color scale bar indicates log2 fold changes of kinase/Cdc37 interactions between inhibitor and DMSO treatments. Synthetic lethality inducing drugs (red), no effect inducing drugs (black). Data analysis was performed using R (R Core Team. R: A Language and Environment for Statistical Computing; https://www.r-project.org/).

Figure S2

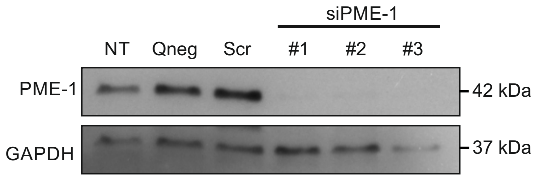

Fig. S2. Immunoblot assessment of PME-1 in T98G cells siRNA kinase screen samples.

Figure S3

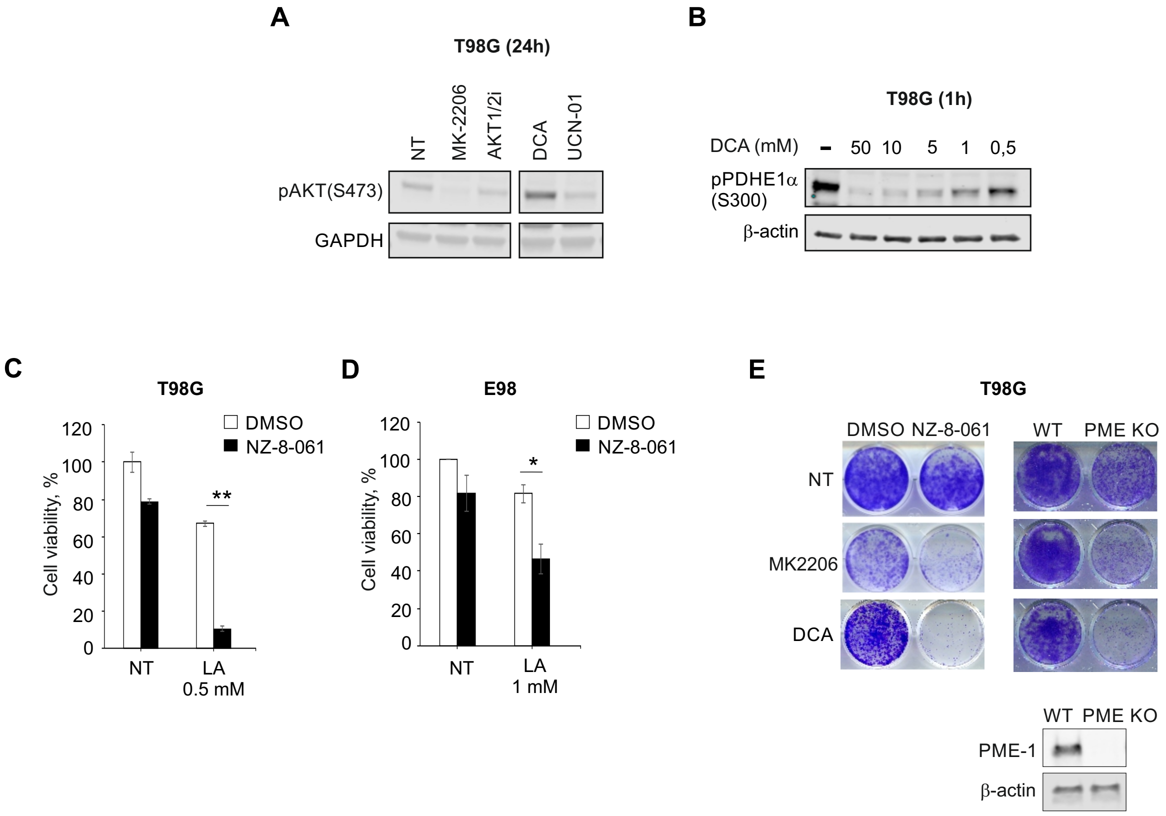

Fig. S3. Hit validation in heterogeneous GB cell lines. A) Immunoblot assessment of phosphorylated AKT (S473) after treatment with 5 μM MK-2206, 10 μM AKT1/2i, 20mM DCA and 25 nM UCN-01 for 24 h in T98G. **B)** IImmunoblot assessment of phosphorylated PDHE1α (S300) after 1 h treatment with DCA at the indicated concentrations. **C-D)** Cell viability in T98G (C) and E98 (D) cells treated with NZ-8-061 in combination of lipolic acid (LA) for 72 h. Results are presented as mean ± SD (n = 3 independent experiments). *P<0.05, **P<0.01 by Student's t-test. **E)** Representative images of colony formation assay in T98G cells under PP2A activation by NZ-8-061 (8 μM) or PME-1 knockdown in combination with 5 μM MK-2206 and 20 mM DCA. Western blot analysis of PME-1 knockdown (lower panel).

Original colony growth assay scans

Fig.2A

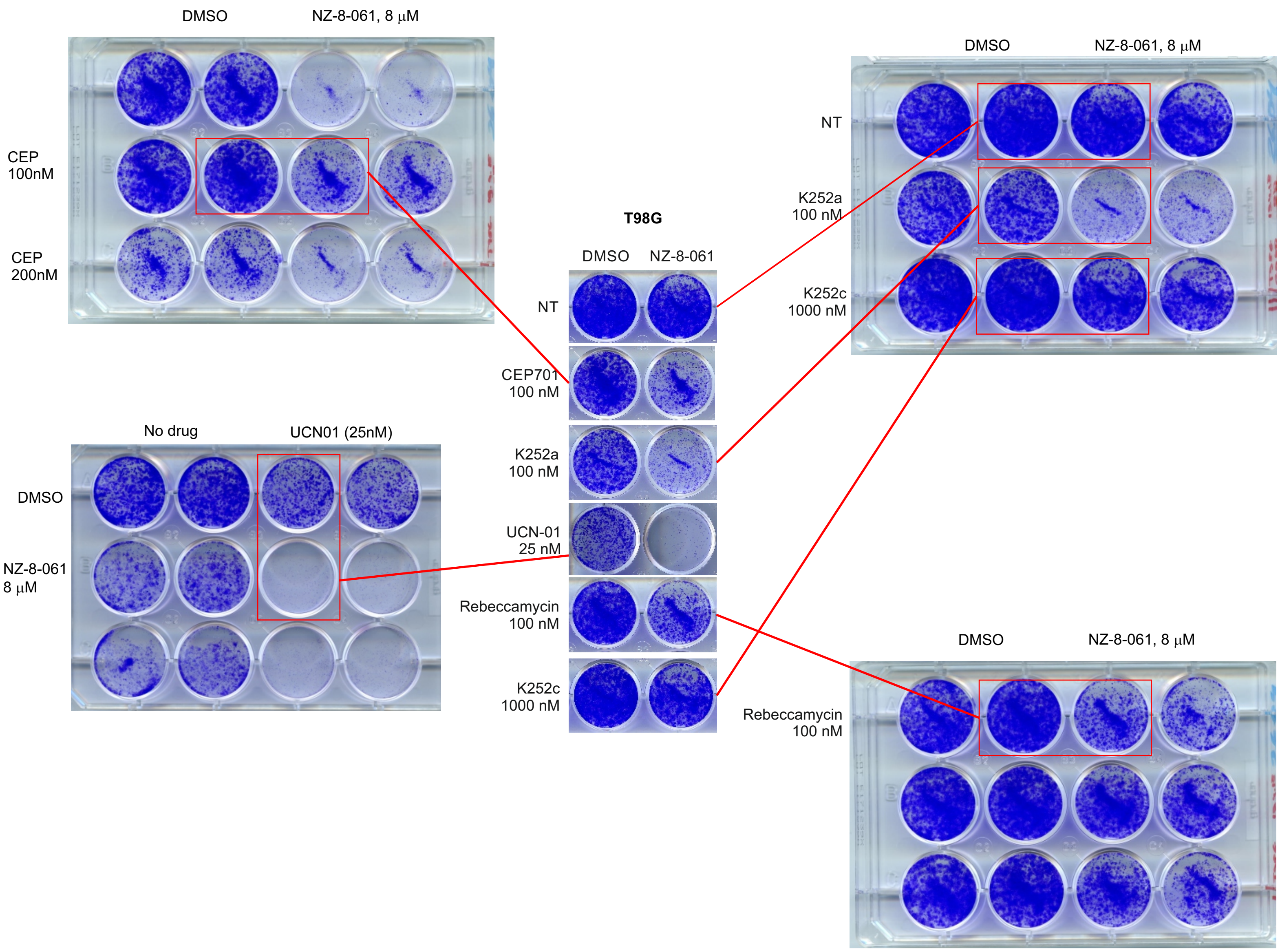

Original colony growth assay scans

Fig.S3E

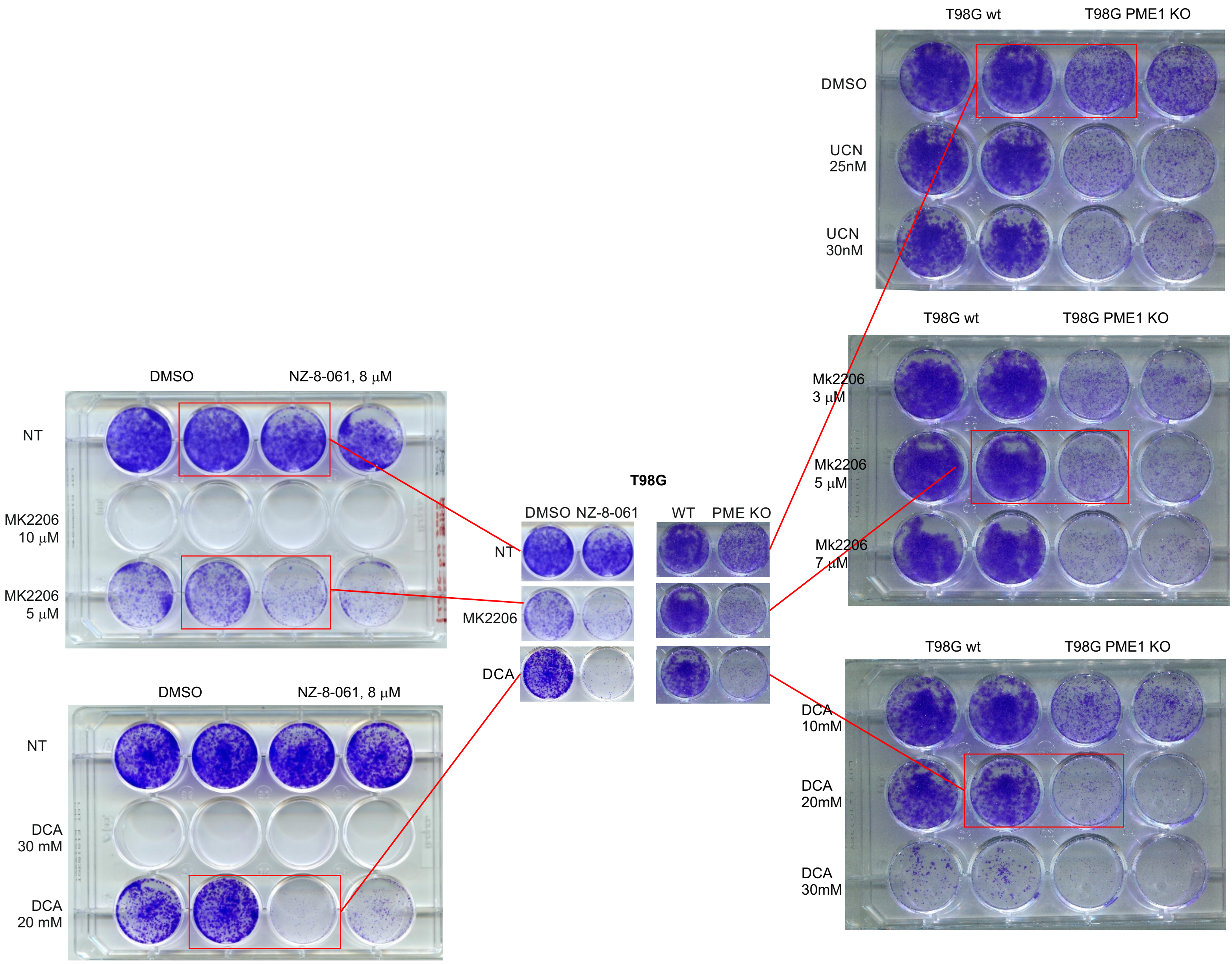

Supplementary Original western blot images

Fig.S2

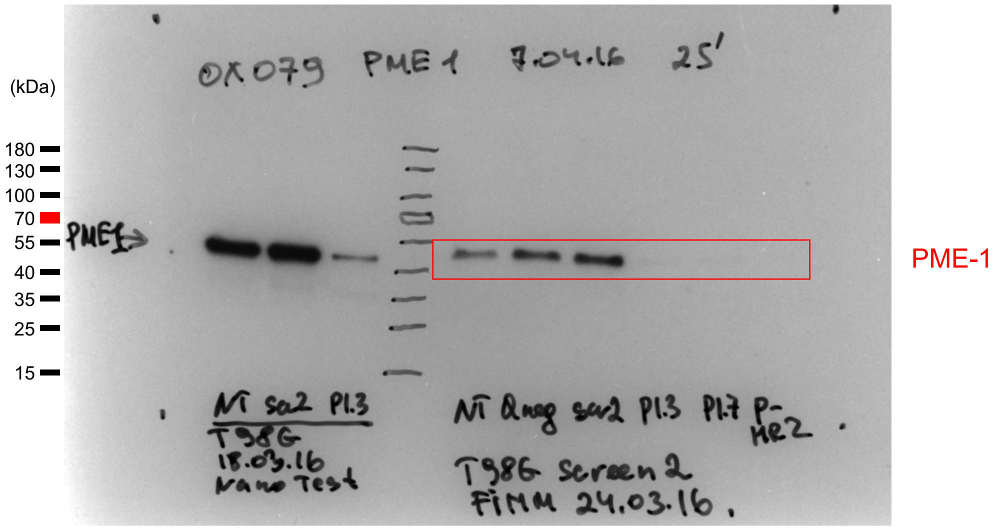

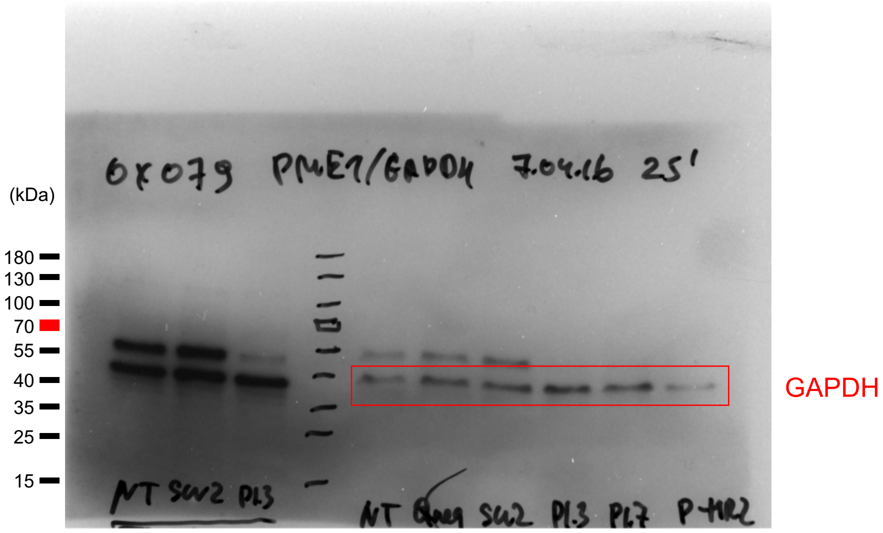

Supplementary Original western blot images

Fig.S3A

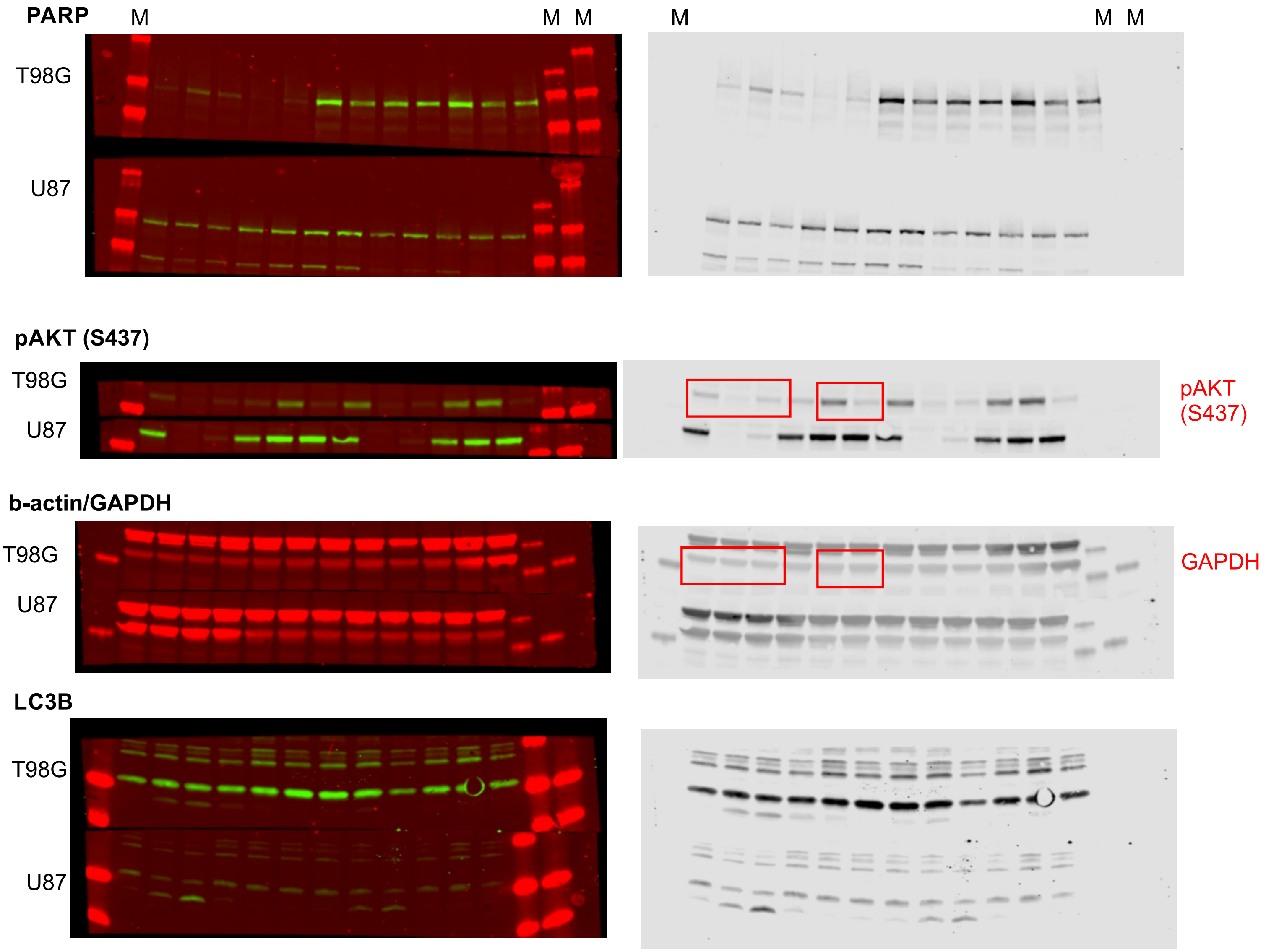

Supplementary Original western blot images

Fig.S3B

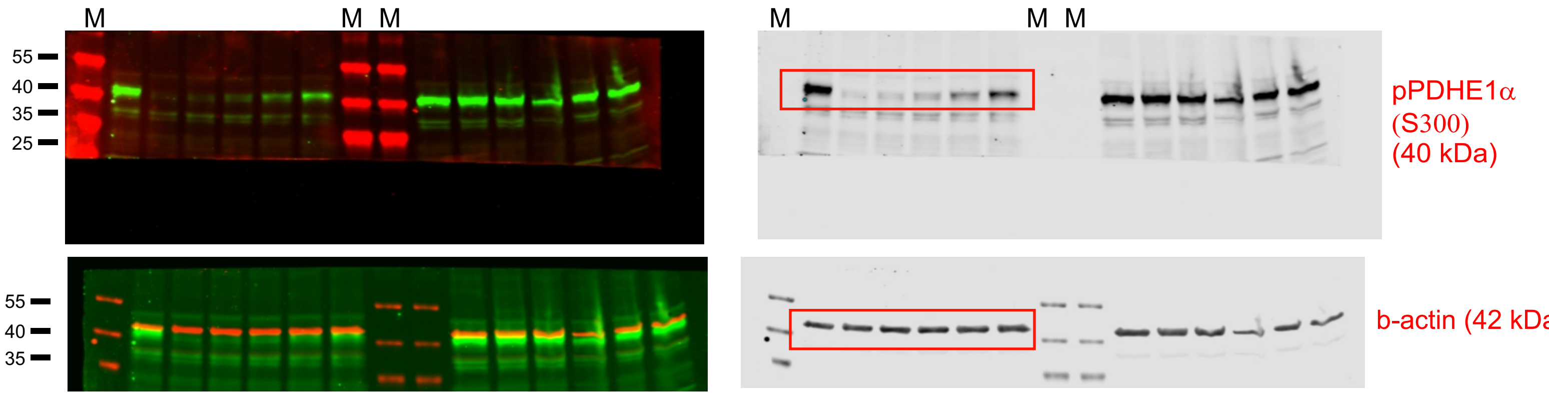

Fig.S3E

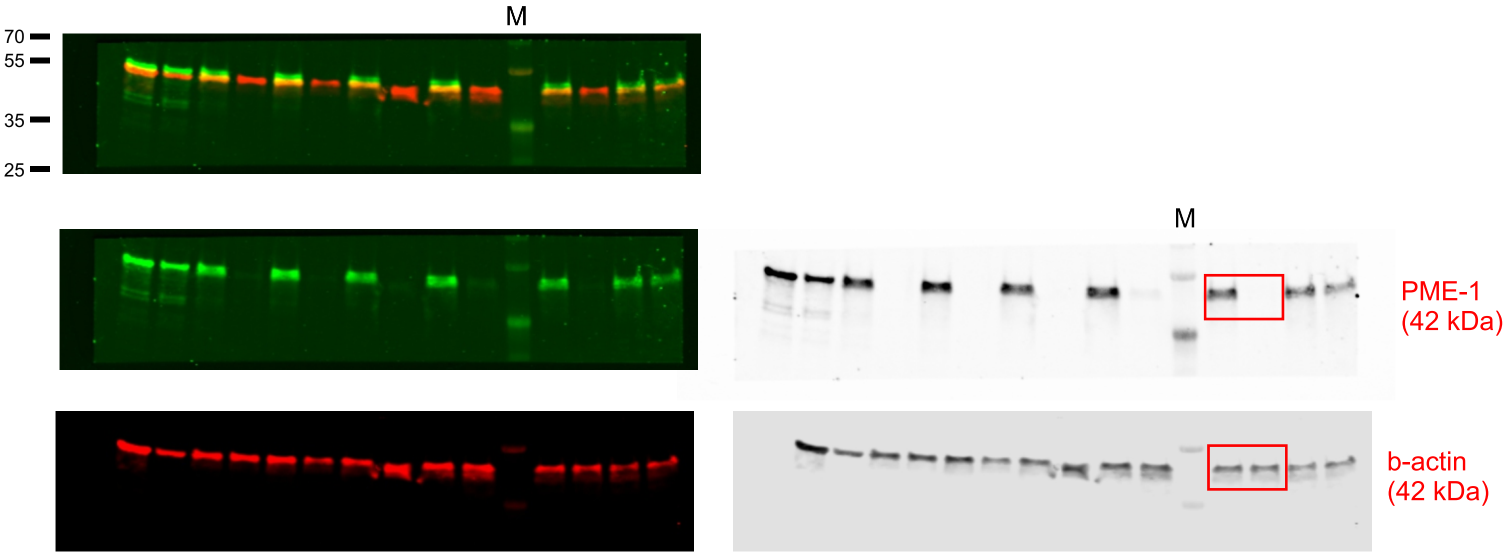

Supplement: Supplementary file 1 — Supplementary Information 1. [file 41598_2022_18118_MOESM1_ESM.pdf]
